# The Corticofugal Effects of Auditory Cortex Microstimulation on Auditory Nerve and Superior Olivary Complex Responses Are Mediated via Alpha-9 Nicotinic Receptor Subunit

**DOI:** 10.1371/journal.pone.0155991

**Published:** 2016-05-19

**Authors:** Cristian Aedo, Gonzalo Terreros, Alex León, Paul H. Delano

**Affiliations:** 1 Programa de Fisiología y Biofísica, ICBM, Facultad de Medicina, Universidad de Chile, Santiago, Chile; 2 Departamento de Otorrinolaringología, Hospital Clínico de la Universidad de Chile, Santiago, Chile; 3 Departamento de Tecnología Médica, Facultad de Medicina, Universidad de Chile, Santiago, Chile; Osaka University Graduate School of Medicine, JAPAN

## Abstract

**Background and Objective:**

The auditory efferent system is a complex network of descending pathways, which mainly originate in the primary auditory cortex and are directed to several auditory subcortical nuclei. These descending pathways are connected to olivocochlear neurons, which in turn make synapses with auditory nerve neurons and outer hair cells (OHC) of the cochlea. The olivocochlear function can be studied using contralateral acoustic stimulation, which suppresses auditory nerve and cochlear responses. In the present work, we tested the proposal that the corticofugal effects that modulate the strength of the olivocochlear reflex on auditory nerve responses are produced through cholinergic synapses between medial olivocochlear (MOC) neurons and OHCs via alpha-9/10 nicotinic receptors.

**Methods:**

We used wild type (WT) and alpha-9 nicotinic receptor knock-out (KO) mice, which lack cholinergic transmission between MOC neurons and OHC, to record auditory cortex evoked potentials and to evaluate the consequences of auditory cortex electrical microstimulation in the effects produced by contralateral acoustic stimulation on auditory brainstem responses (ABR).

**Results:**

Auditory cortex evoked potentials at 15 kHz were similar in WT and KO mice. We found that auditory cortex microstimulation produces an enhancement of contralateral noise suppression of ABR waves I and III in WT mice but not in KO mice. On the other hand, corticofugal modulations of wave V amplitudes were significant in both genotypes.

**Conclusion:**

These findings show that the corticofugal modulation of contralateral acoustic suppressions of auditory nerve (ABR wave I) and superior olivary complex (ABR wave III) responses are mediated through MOC synapses.

## Introduction

The auditory efferent system is a complex network of descending pathways, which are mainly originated from pyramidal neurons located in layers V and VI of the primary auditory cortex [1–4], and are directed to the auditory thalamus [5], inferior colliculus [6, 7], cochlear nucleus [8–10], and even directly to the superior olivary complex (SOC) [11]. These descending pathways are directly or indirectly connected -through inferior colliculus synapses- to olivocochlear neurons [11–12], which are located in the SOC and constitute the final pathway to the cochlear receptor and auditory nerve. Based on the anatomic origin and in the morphological characteristics of olivocochlear neurons, these can be classified into two groups: the medial olivocochlear system (MOC), which makes synapses with outer hair cells (OHC) and the lateral olivocochlear system (LOC), which innervates type-I auditory nerve neurons [13]. MOC neurons release acetylcholine and activate nicotinic receptors composed by α9 and α10 subunits, which are specific for MOC synapses, as these receptors are not expressed in LOC synapses [14, 15].

Physiologically, the olivocochlear function can be evaluated using contralateral acoustic stimulation at moderate sound pressure levels, which suppresses auditory nerve and cochlear responses [16, 17]. The neural circuit of this reflex is located in the brainstem and comprises ipsilateral auditory nerve fibers, ipsilateral cochlear nucleus neurons, and contralateral uncrossed MOC fibers [18]. Recently, we demonstrated in chinchillas that the electrical microstimulation of the auditory cortex modulates the effects of contralateral acoustic stimulation on auditory nerve responses [19]. Specifically, the auditory cortex microstimulation (ACMS) overrides the strength of this acoustic reflex to an optimal amplitude suppression of around 1 dB, meaning that large reductions were diminished by cortical microstimulation, while small suppressions were enhanced by ACMS. Importantly, these corticofugal effects were only observed when evaluating auditory nerve responses but not cochlear microphonics (a measure of OHC function). Therefore, the corticofugal effects of ACMS observed in chinchillas [19] could have been produced by a direct modulation of LOC activity or indirectly through a modulation of MOC neurons.

In the present work, we tested the proposal that the corticofugal effects that modulate the strength of the olivocochlear reflex on auditory nerve responses are produced through cholinergic synapses between MOC neurons and OHC via alpha-9/10 nicotinic receptors. Wild type (WT) and alpha-9 nicotinic receptor (α9-nAChR) knock-out (KO) mice, which lack cholinergic transmission between MOC and OHC [20], were used to evaluate the consequences of ACMS in the effects produced by contralateral acoustic stimulation on auditory brainstem responses (ABR). The ABR technique allowed us to record afferent responses from different levels of the auditory pathway, including auditory nerve (wave I), superior olivary complex (wave III) and inferior colliculus (wave V). We found that the descending effects of ACMS on ABR waves I and III were absent in the α9-nAChR KO mice, suggesting that the corticofugal effects of ACMS on auditory nerve and superior olivary complex responses are produced through MOC synapses.

## Methods

### Animals

Data were obtained from twelve WT and nine α9-nAChR KO adult mice of either sexes, aged between 60 and 150 days and weighing between 20 and 35 g. WT and α9-nAChR KO mice on background CBA/129SvEv [20] were kindly provided by Dr. Douglas Vetter from the University of Mississippi, USA. Genotypes were confirmed before and after the surgical procedures by PCR screening of genomic DNA extracted and purified from the tail. All procedures were approved by the local committee of Bioethics (Comité de Bioética Animal, permit number #0452, Facultad de Medicina, Universidad de Chile) and were performed in accordance to the Guidelines for the Care and Use of Laboratory Animals (publication number 86–23 National Institute of Health, revised 1996). At the end of the experiments, two mice were sacrificed and perfused intracardially with 0.9% saline solution followed by 4% paraformaldehyde for histological processing of the brain, while the rest of the mice were euthanized using isoflurane overdose followed by cervical dislocation.

### Surgical procedures

Mice were anesthetized with xylazine (10 mg/kg I.P.), ketamine (100 mg/kg, I.P.), and atropine (0.1 mg/kg, I.P.). In addition, half doses of ketamine were given every 45–60 minutes depending on paw withdrawal reflex. Mice were placed in a stereotaxic device (SR-6M, Narishige International^®^, NY, USA) and body temperature was maintained at 37–38°C by means of a heating pad. Under general anesthesia, a sagital scalp midline incision was made, exposing the skull. Subsequently a small craniotomy on the left temporal lobe, located by stereotactic coordinates was performed [21] (from 2.2 to 3.6 mm posterior to bregma, 4.0 to 4.5 mm lateral to the midline). After the dura mater was exposed, we proceeded to position a Nichrome^®^ stereotrode on the cortical surface (a pair of low impedance (<5 kΩ) electrodes (200 μm) separated by 400 μm). The dura mater was minimally sectioned to facilitate the penetration of the pair of electrodes to a cortical depth of 800 μm, which allowed the recording of evoked responses and the electrical microstimulation of deep cortical layers (V and VI). As previous evidence in rats has shown that auditory cortex descending projections to subcortical nuclei are mainly originated from primary auditory cortex [22], we searched for electrode positions that had short auditory cortex evoked potential (ACEP) latencies (< 14 ms), compatible with primary auditory cortex locations in mice [23]. In addition, in two mice, we performed histological confirmation (Nissl staining) of the electrode depth and location used in all animals. Ground and reference electrodes were inserted at the midline of the mice cranium.

### Auditory stimuli

Ipsilateral tones (right ear, 15 kHz at 80 dB SPL) and contralateral broadband noise (CBN) (left ear, 55–60 dB SPL) were digitally generated by two synchronized multifunction computer boards at 100,000 samples/s (PCI-6071-E, National Instruments^®^, TX, USA), attenuated by PA-5 programmable attenuators (system III, Tucker Davis Technologies^®^, FL, USA). We decided to use 15 kHz tones due to evidence of a higher density of olivocochlear innervation at this position of the cochlea in mice [24]. Auditory stimuli were delivered via two tweeters (one for ipsilateral and the second for contralateral auditory stimuli) (Realistic super tweeter, frequency response 5–40 kHz, Radioshack^®^, TX, USA) through tubes sealed to the external auditory meatus. Ipsilateral tones were presented with alternating polarity at 4 Hz rate, 5 ms duration, and 0.5 ms rise/fall time and were used to obtain ipsilateral auditory brainstem responses and contralateral ACEP. Contralateral non-continuous broadband noise (5–40 kHz) was presented at 4 Hz with a duration of 170–200 ms.

### Electrophysiological procedures

All electrophysiological procedures were conducted by one experimenter blinded to the genotype of the animals. In every experiment, two different electrophysiological recordings were obtained: (i) auditory cortex evoked potentials and (ii) auditory brainstem responses. In addition, cortical stereotrodes were used to perform (iii) electrical microstimulation during simultaneous acquisition of ABR responses.

#### (i) Auditory cortex evoked potentials

The electrical signals from the electrodes positioned in the left auditory cortex were amplified 10,000X and filtered between 1 and 1000 Hz, using a BMA-200 differential preamplifier (Cwe-inc^®^, PA, USA) and in a second stage low-pass filtered at 200 Hz (model 901, Frequency devices^®^, IL, USA). Signals were acquired and digitized at 40,000 samples/s with a multifunction acquisition board (PCI-6071-E, National Instruments^®^, TX, USA).

#### (ii) Auditory brainstem responses

ABRs signals were acquired through intradermal needle electrodes (low impedance (< 5 kΩ), 25G needle diameter) directed to the ear canal of both ears. Ground electrodes were placed in the midline of the animal [25]. The brainstem signal in response to ipsilateral stimulation (right ear) was amplified 10,000–100,000 times and filtered between 0.3 and 3 kHz. Data were digitized and stored for offline analyses at 40,000 samples per second using a multifunction acquisition card (PCI-6071-E, National Instruments^®^, TX, USA) and a custom made software programmed in C language (LabWindows CVI 6.0, National Instruments, TX, USA).

#### (iii) Auditory cortex electrical microstimulation

Trains of four biphasic square electrical pulses (1–4 μA, 0.25 ms each, separated by 2.2 ms) were delivered at 32 Hz rate to the auditory cortex during 5 min, using an isolated pulse generator (model 2100, AM-Systems^®^, WA, USA). This protocol was based on previous experiments of corticofugal effects of auditory cortex microstimulation performed in bats and chinchillas [19, 26]. In order to avoid electrical artifacts generated by cortical microstimulation, ipsilateral and contralateral auditory stimuli were presented with a time delay of 10 to 15 ms. However, in two KO mice, wave I effects were eliminated from analyses due to masking by ACMS artifacts.

### Experimental protocol

**Fig 1:**
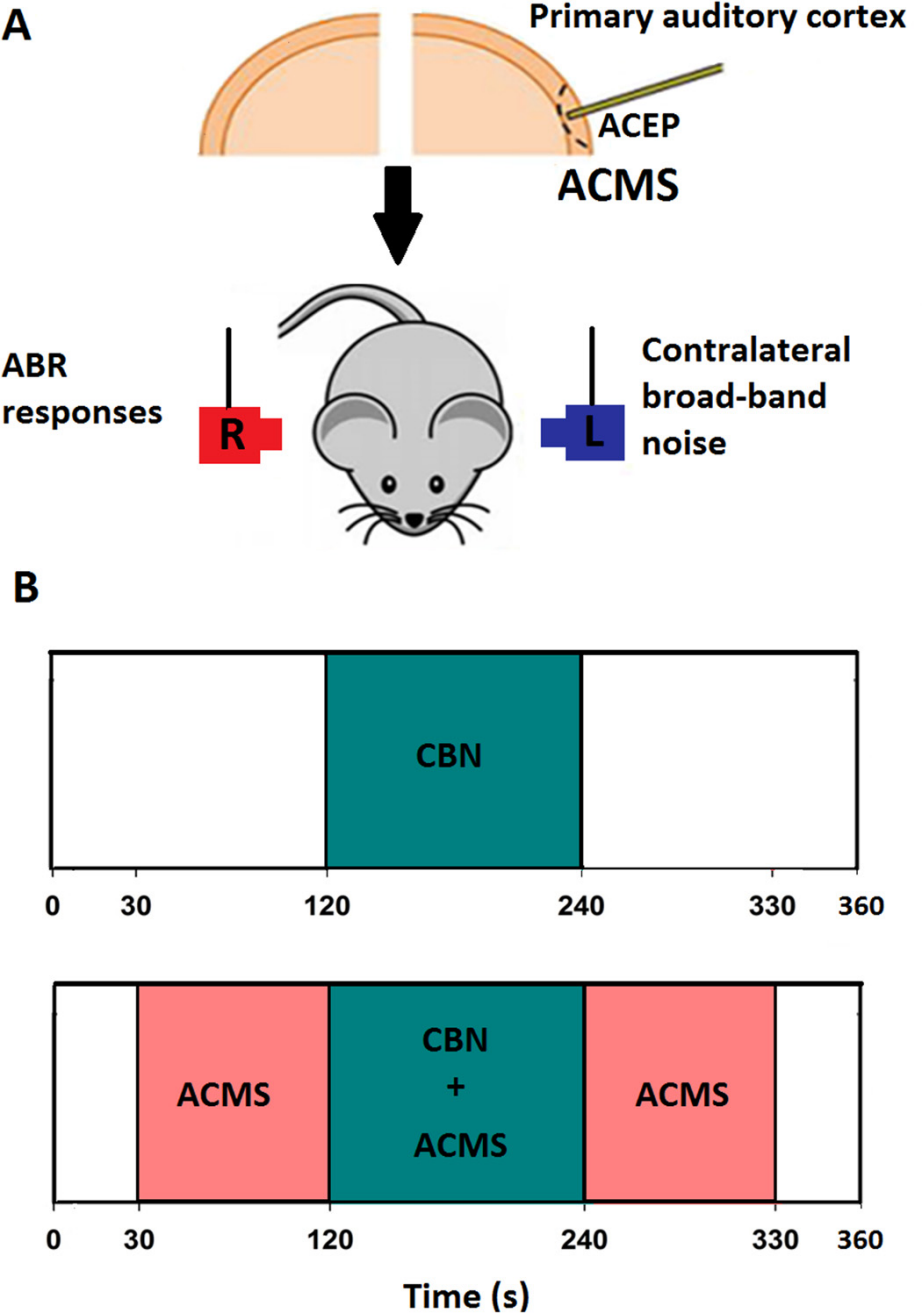
Schematic diagram of the experimental protocol used in WT and alpha-9-KO mice. A. In order to record auditory cortex evoked potentials (ACEP), a stereotrode was positioned in the left auditory cortex. This electrode was also used to perform the auditory-cortex electrical microstimulation (ACMS). Contralateral broad-band noise (CBN) was presented in the left pinna, while ipsilateral 15 kHz tones at 80 dB SPL in the right pinna. B. Temporal course of the experimental protocol. The effects of CBN were evaluated without (upper panel) and with ACMS (bottom panel). Auditory-brainstem responses (ABR) elicited by 15 kHz tone bursts were measured throughout the experimental protocol at 4 Hz rate. The period of contralateral acoustic stimulation is illustrated in green (120 to 240 s), while the ACMS period is depicted in pink (30 to 330 seconds).

Fig 1 illustrates the time course of the experimental protocol. For each recording, two protocols, with and without ACMS were performed. In the first protocol, without ACMS, ipsilateral tones were presented without contralateral stimulation for two minutes, while between minutes 2 and 4, we delivered simultaneously CBN. Finally, during minutes 4 to 6, ipsilateral tones were presented without contralateral stimulation. This six minute protocol was repeated with ACMS, delivered between 30 and 330 seconds of the protocol (Fig 1B).

### Data analyses

Averaged ACEP waveforms were obtained before the ACMS protocol from 1440 trials. The effects of CBN stimulation on the amplitudes of ABR waves was evaluated with and without ACMS in WT and KO mice. The amplitude change of wave I produced by CBN was chosen as a measure of the strength of the MOC reflex on auditory nerve responses. On the other hand, amplitudes changes of ABR waves III and V were also quantified, and were considered as measures of the effects of CBN on superior olivary complex and inferior colliculus responses respectively. The effects of CBN alone, and of CBN with ACMS on the amplitudes of ABR waves (I, III, and V) were calculated in dB referenced to the corresponding wave amplitude without CBN and without ACMS: (dB amplitude suppression = 20*Log_10_[wave amplitude with CBN (or ACMS)/wave amplitude without CBN and ACMS]).

### Statistical analyses

Normal distribution of data were evaluated using Shapiro-Wilk tests, and analyzed accordingly with parametric or non-parametric statistical tests. Latencies differences between WT and KO mice were evaluated using Mann-Whitney Rank Sum Test. For grand average analyses and statistical effects of ABR amplitudes, a single ABR per animal and per condition (ACMS and genotype) were used. In these data, the effects of CBN on ABR waves in WT and KO mice in the presence and absence of ACMS were evaluated using a two-way analysis of variance (ANOVA) and Bonferroni post-hoc tests. Correlations between ACEP latencies and corticofugal effects of ACMS and CBN were evaluated with Spearmen tests. In the temporal course analysis, twelve averaged ABRs obtained each 30 seconds (120 repetitions) of the experimental protocol were analyzed with a two-way repeated measures ANOVA and Bonferroni post-hoc tests. A p value <0.05 was considered as significant in all statistical tests. Data set used in graphs is available in S1 File.

## Results

**Fig 2:**
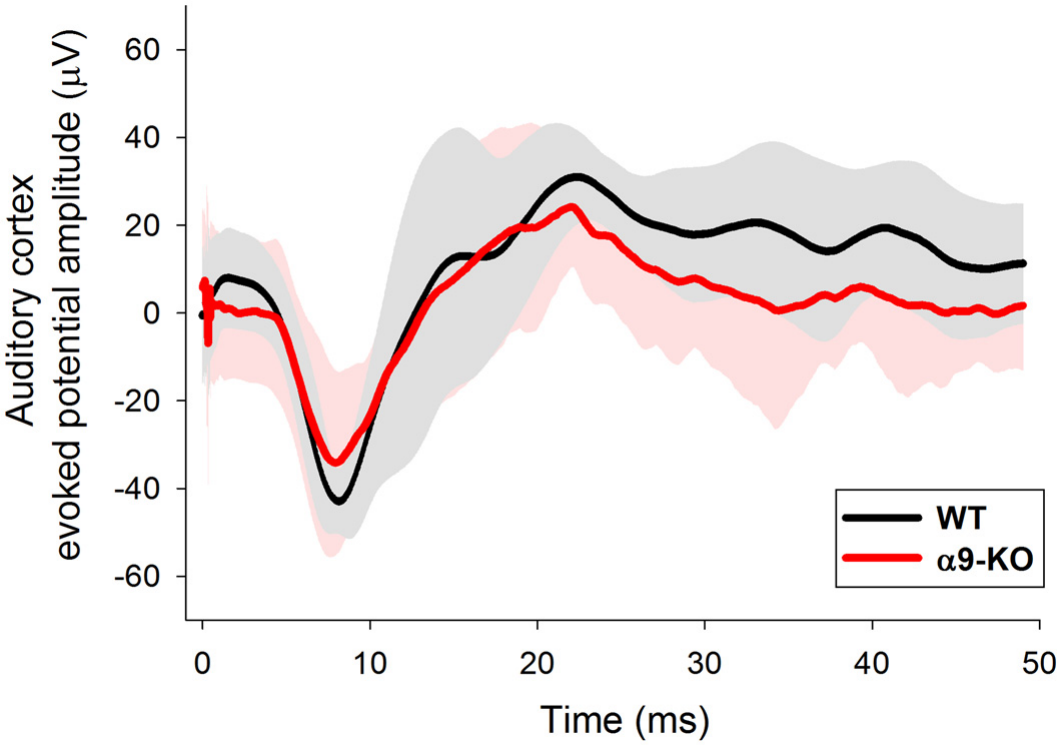
Auditory-cortex evoked potentials in WT and alpha-9-KO mice are similar. Grand average ACEPs obtained with 15 kHz tones delivered at 80 dB SPL in WT and alpha-9-KO mice. Note the latency and amplitude similarity of the ACEPs obtained in WT and alpha-9-KO mice. Gray and pink shaded areas represent the range of two standard deviation ± the mean of WT and alpha-9-KO mice correspondingly.

We recorded ACEP responses in 21 animals, including twelve WT and nine alpha-9-KO mice. There were no differences in the latencies of ACEPs between WT and KO mice (Fig 2). The mean latency of the first negative peak of ACEP responses for WT animals (n = 12) was 9.24 ± 1.83 ms (mean ± standard deviation) while for KO mice (n = 9) was 8.53 ± 1.98 ms, (Mann-Whitney Rank Sum Test, U = 36.0, p = 0.213, non-significant difference), while for the second positive peak, mean latencies were 26.76 ± 6.47 ms for WT animals, and 23.92 ± 8.25 ms for KO mice (Mann-Whitney Rank Sum Test, U = 40.5, p = 0.355, non-significant difference).

**Fig 3:**
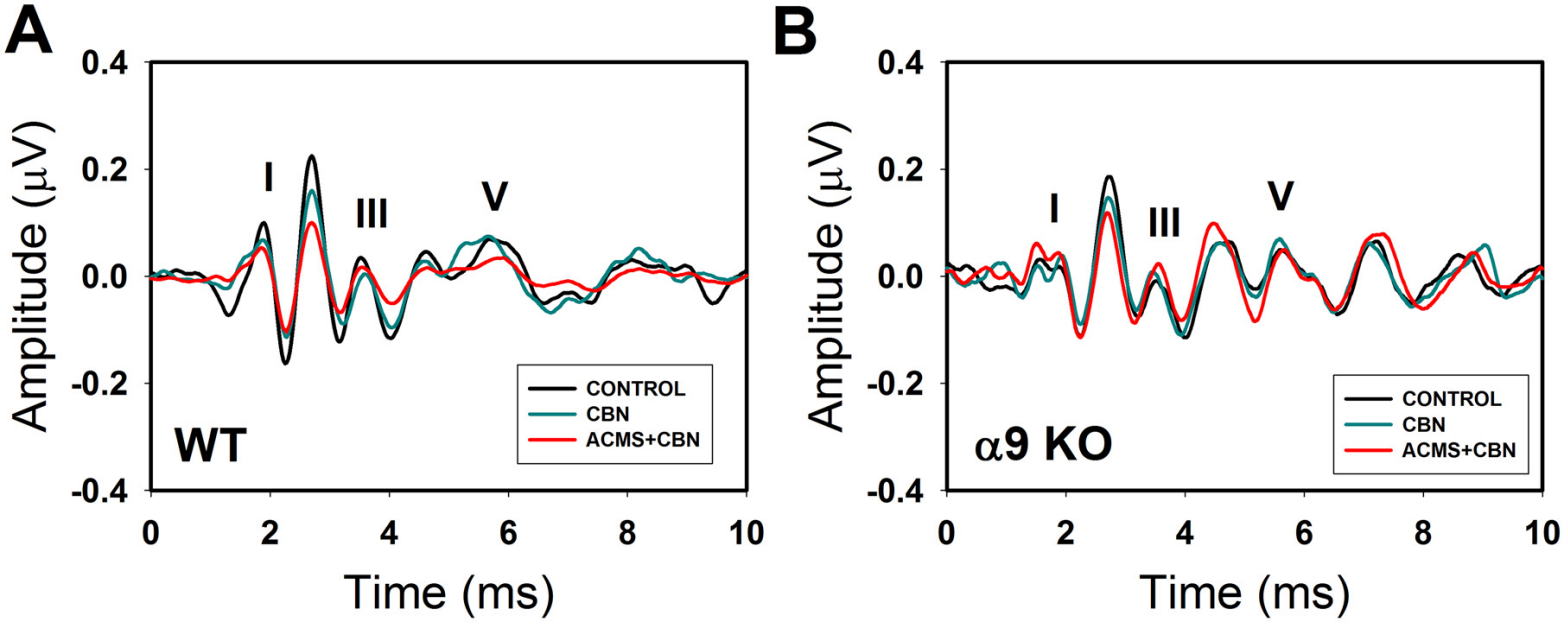
Contralateral broad-band noise and ACMS produce larger reductions of ABR waveforms in WT than in alpha-9-KO mice. This figure shows examples of ABR obtained in WT and alpha-9-KO mice in three different conditions: control (black trace), with contralateral broad-band noise (green trace) and with CBN and ACMS (red trace). **A**. Note that the amplitudes of the ABR-waves (I, III and V) are clearly reduced in the WT mice, while smaller changes of ABR waves were observed in the alpha-9-KO mice.

**Fig 4:**
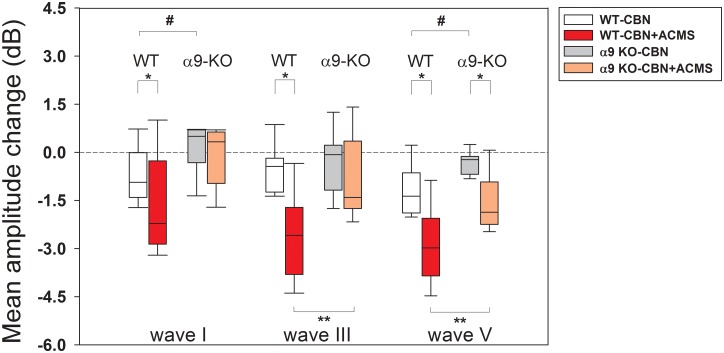
Auditory-cortex electrical microstimulation enhances the suppressive effects of contralateral noise on ABR waves I and III in WT mice, but not in alpha-9-KO mice. This figure shows a summary of average corticofugal effects in all studied animals. Box-plots display the median and interquartile distribution of the effects of CBN in WT and alpha-9-KO mice without and with ACMS. The reference level for calculating amplitude changes in dB were the ABR amplitudes obtained without noise and without ACMS. Significant differences between WT and KO mice were obtained for CBN stimulation compared to the no noise and no ACMS condition for waves I and V (CBN effects: #p<0.05; two-way ANOVA). Significant differences were obtained for the effects of ACMS in WT mice for waves I, III and V and for wave V in alpha-9-KO mice. In addition, the effects of ACMS on wave III and V were larger for WT than for KO mice (genotype effects: *p<0.05; ACMS effects: **p<0.05; two-way ANOVA).

After ACEP recordings were performed, we evaluated the effects of CBN on ABR responses (ABR waves I, III and V) with and without ACMS in WT and alpha-9-KO mice. Fig 3 shows illustrative examples of ABR waves obtained in WT and KO mice in three conditions: (i) without CBN and ACMS, (ii) with CBN and without ACMS, and (iii) with CBN and ACMS. Note that in this illustrative example, CBN produces reductions in the amplitudes of ABR waves in WT mice that were enhanced after ACMS. On the other hand, ABR amplitudes were not significantly affected by CBN and ACMS in the alpha-9-KO mice. Fig 4 shows grand average effects of CBN with and without ACMS in WT and alpha-9-KO mice. A two-way ANOVA was used to determine the effects of genotype (WT vs alpha-9-KO) and presence of ACMS on the strength of the olivocochlear reflex, measured as amplitude reduction (in dB) of ABR waves I, III and V with CBN. A significant effect of genotype was found for amplitude reduction of wave I (F_(1,34)_ = 10.449, p = 0.003), but not for ACMS (F_(1,34)_ = 2.625, p = 0.114). Bonferroni post-hoc tests showed significant differences of CBN effects (without ACMS) between WT and KO mice on wave I (t = 2.259, #p = 0.03), significant effects of CBN and ACMS on wave I for WT mice (t = 2.173, *p = 0.037), but not for KO mice (t = 0.379, p = 0.707). Regarding wave III, we found a significant reduction for genotype F_(1,38)_ = 8.133, p = 0.007), and for ACMS (F_(1,38)_ = 13.384, p<0.001). Bonferroni post-hoc tests showed no difference of CBN effects (without ACMS) between WT and KO mice on wave III (t = 0.422, p = 0.676), significant effects of ACMS on wave III for WT mice (t = 4.517, *p<0.001), but not for KO mice (t = 0.928, p = 0.359). Finally, a significant effect of genotype was found for amplitude reduction of wave V (F_(1,38)_ = 16.861, p<0.001) and for ACMS (F_(1,38)_ = 30.897, p<0.001). Bonferroni post-hoc tests showed significant differences of CBN effects (without ACMS) between WT and KO mice on wave V (t = 3.233, #p = 0.003), significant effects of ACMS on wave V for WT mice (t = 4.579, *p<0.001), and for KO mice (t = 3.074, *p = 0.004).

**Fig 5:**
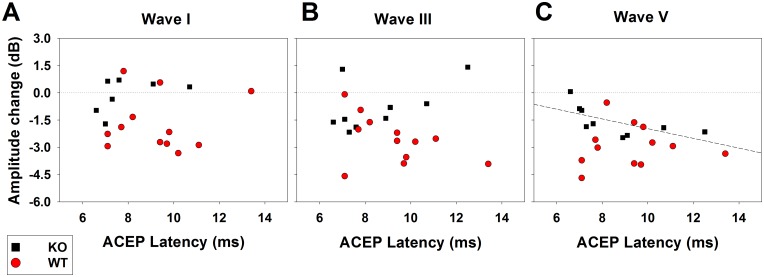
Relation between the magnitude of ACMS corticofugal effects on ABR responses and ACEP latencies. Black squares and red circles correspond to the amplitude changes of individual ABR responses with ACMS and CBN in the KO and WT mice correspondingly (A: wave I; B: wave III; and C: wave V). All these corticofugal effects were obtained with ACEP latencies below 14 ms, which are suggestive of primary auditory cortex recording sites. Notice that the only significant correlation was between the latency of ACEP and the amplitude effects on wave V in the KO mice (Spearman correlation index: -0.833, p = 0.002), which suggest that longer ACEP latencies produce larger corticofugal effects on the inferior colliculus in KO mice.

As neuroanatomical studies have shown that most corticofugal projections are originated in the primary auditory cortex [22], and as there is physiological evidence that cortical sites with shorter ACEP latencies have larger corticofugal effects on auditory nerve responses [19], we studied possible differences related to ACEP latencies in WT and KO mice. Fig 5 shows that there were no significant correlations between ACEP latencies and the corticofugal effects of ACMS and CBN in WT and KO mice for ABR waves I and III. On the other hand, a significant negative correlation (Spearman test, p<0.05) was found for ACEP latencies and corticofugal effects on wave V in KO mice.

**Fig 6:**
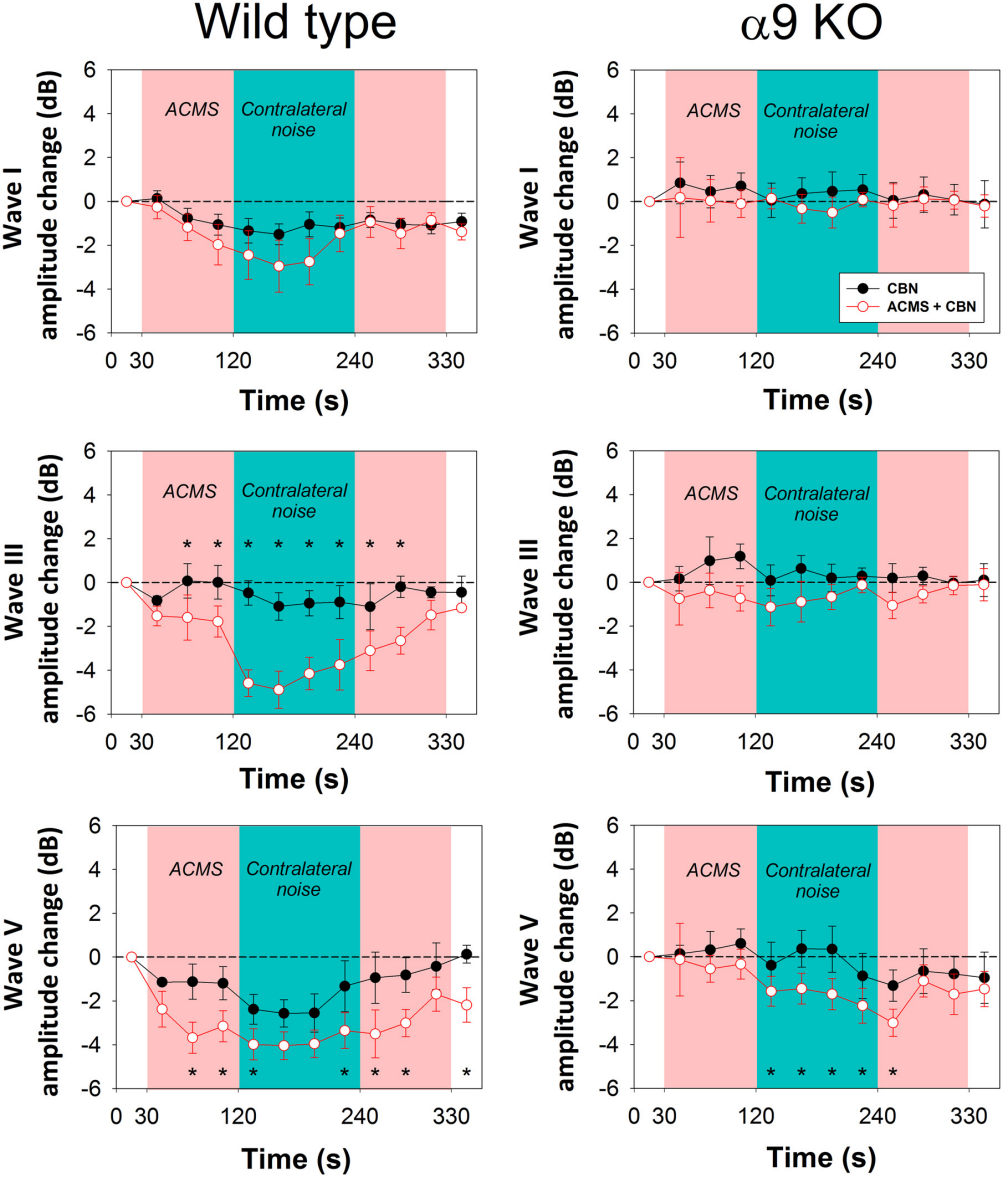
Temporal course of corticofugal effects produced by ACMS on ABR waves in WT and alpha-9-KO mice. The grand averages of ABR obtained each 30 seconds of the protocol (a total 12 epochs per condition, each one computed from 120 responses) are displayed. Black circles represents ABR waves measured without ACMS, while red border white circles represent ABR waves obtained with ACMS. CBN were presented in both conditions between 120 and 240 seconds of the protocol. Asterisks represent significant effects of ACMS in different epochs of the experimental protocol (*p<0.05, repeated measures ANOVA, Bonferroni, post-hoc test). In addition, there were significant differences in the ABR wave-I amplitudes in the different epochs in WT (epoch 2 compared to epochs 5, 6 and 7 during simultaneous presentation of ACMS and CBN).

Next, in order to evaluate the temporal course of corticofugal effects produced by ACMS on ABR waves, we analyzed ABR recordings averaged each 120 repetitions (30 seconds). As each recording of the experimental protocol was designed to be completed in 6 minutes, each experimental session was divided into 12 epochs along time. The number of 120 averaged trials was the lowest number that allowed us to identify ABR waves for reliable measurements. Fig 6 shows the grand average of the temporal course of the effects of ACMS and CBN for ABR waves I, III and V in WT and alpha-9-KO mice. A two-way repeated measures ANOVAs for ABRs waves, considering the temporal course (12 epochs) and the presence of ACMS as factors revealed significant effects of the temporal course for waves I, III and V, and significant effects of ACMS for waves III and V in WT mice (wave I: Temporal course: F_(10,110)_ = 3.061, p = 0.002; ACMS: F_(1,11)_ = 0.306, p = 0.591. Wave III: Temporal course: F_(10,110)_ = 7.806, p<0.001; ACMS: F_(1,11)_ = 17.003, p = 0.002. Wave V: Temporal course: F_(10,110)_ = 6.727, p<0.001; ACMS: F_(1,11)_ = 16.590, p = 0.002). On the other hand in KO mice, we only found significant effects for the temporal course and ACMS for wave V, but no effects were obtained for waves I and III in the KO mice (wave I: Temporal course: F_(6,60)_ = 1.904, p = 0.062; ACMS: F_(1,6)_ = 1.695, p = 0.241. Wave III: Temporal course: F_(8,80)_ = 0.779, p = 0.648; ACMS: F_(1,8)_ = 4.385, p = 0.070. Wave V: Temporal course: F_(8,80)_ = 7.345, p<0.001; ACMS: F_(1,8)_ = 25.683, p<0.001).

Notice that the temporal course analysis evidenced the presence of direct effects of ACMS on the amplitudes of ABR waves in WT mice (without CBN). These corticofugal effects correspond to the significant changes in the amplitudes of waves III and V observed in the time epochs between 30 and 120 seconds of the experimental protocol in WT mice (Fig 6). On the other hand, no direct effects of ACMS (without CBN) were observed in any ABR wave in the alpha-9-KO mice.

## Discussion

In the auditory system, descending pathways from the auditory cortex to subcortical nuclei form multiple afferent-efferent circuits, including cortico-thalamic, cortico-collicular, cortico-superior olivary complex and cortico-cochlear nucleus [3, 4, 27]. Physiological studies performed in rodents show that the deactivation, lesion or electrical microstimulation of the auditory cortex produced corticofugal modulations in the afferent responses of the inferior colliculus [28, 29], cochlear nucleus [30, 31], auditory nerve [19, 32] and cochlear responses [33, 34]. Here we used the ABR technique to record afferent responses from different levels of the auditory pathway, including auditory nerve (wave I), superior olivary complex (wave III) and inferior colliculus (wave V) [35]. Moreover, we assessed the effects of CBN on ABR waves in WT and alpha-9-KO mice with and without ACMS. This experimental protocol allowed us to evidence those corticofugal effects that directly depend on MOC-OHC synapses (wave I and III) from the effects on wave V, which were only partially affected in alpha-9-KO mice.

### No differences in ACEP responses between WT and alpha-9-KO mice

The present work provides original data showing no differences in auditory cortex evoked potentials between WT and alpha-9-KO mice at 15 kHz (Fig 2). These results show that cortical processing of afferent responses at 15 kHz in both genotypes is similar. However, it is important to limit these results to this single frequency assessed in quiet conditions. For instance, during development, Clause and collaborators [36], found broader brainstem tonotopic maps in alpha-9-KO than in WT mice, while Lauer and May [37] and May et al. [38], found that WT and alpha-9-KO mice have comparable ABR responses in quiet conditions, but different ABR responses in the presence of noise or with shortened inter-stimulus intervals. Future work should address whether these brainstem deficits in tonotopic maps [36] and in afferent responses in noise conditions [37, 38] are also present in auditory cortex evoked responses in alpha-9-KO mice.

### Direct effects of ACMS (without CBN) on ABR waves in WT mice

Previous work in chinchillas suggested the presence of a cortico-olivocochlear basal tone of activity that regulates cochlear responses [33]. In that work, pharmacological deactivation and cooling of the auditory cortex produced changes in the amplitudes of auditory nerve and cochlear microphonics potentials. Similar findings have been obtained measuring distortion product otoacoustic emissions during auditory cortex inactivation with lidocaine in gerbils [34]. Moreover, Lamas and collaborators [32], found an increase of ABR thresholds after lesioning the auditory cortex of rats, which was compensated one week after the cortical lesion. In another work, this group of investigators found evidence to propose that the auditory-cortex descending projections regulate the expression of prestin—the OHC protein responsible for cochlear amplification- by modulating MOC synapses [39]. In the present work, ABR responses obtained between 30 and 120 seconds of the experimental protocol (see Fig 6) allowed us to study the corticofugal effects of ACMS without CBN on ABR waves. The results show that ACMS alone modulates the amplitudes of ABR waves III and V in WT but not in alpha-9-KO mice, suggesting that part of the corticofugal effects of ACMS without CBN on subcortical nuclei observed in WT mice are mediated by MOC synapses.

### Effects of CBN on ABR waves I and III are modulated by ACMS in WT but not in alpha-9-KO mice

Contralateral broadband noise at moderate sound pressure levels activates the olivocochlear reflex, which suppresses cochlear and auditory nerve responses [17]. The neural circuit of this reflex is located in the brainstem [18], and the magnitude of its suppressive effect is modulated by descending projections from the auditory cortex [19]. In the present work, we found that ACMS produces an enhancement of CBN suppression of ABR waves I and III in WT mice but not in alpha-9-KO mice (Figs 4 and 6). These results confirm that the corticofugal effects of ACMS on the olivocochlear reflex strength are mediated via alpha-9 nicotinic receptor subunits located in MOC-OHC synapses. However, these results are in partial disagreement with previous data obtained in chinchillas [19], as in that work, the olivocochlear reflex strength was set to an optimal suppression level of about 1 dB, while in the present work in mice, we found that ACMS produced only enhancements of the suppressive effect. This difference could be attributed to species-specific characteristics suggesting that an optimal suppression level of the olivocochlear reflex in mice might be larger than in chinchillas.

Neuroanatomical evidence in rodents has shown that in the auditory system, the majority of the corticofugal projections to subcollicular nuclei are originated in primary auditory cortex [22]. In our experiments, we searched for cortical sites with ACEP latencies shorter than 14 ms (Fig 5), which are compatible with primary fields in mice [23]. Consequently, the corticofugal effects obtained on wave I and wave III by ACMS are most likely produced by corticofugal projections from the primary auditory cortex. One additional argument that supports this statement was the lack of correlation between the corticofugal effects on wave I and III in WT and KO mice and ACEP latencies (Fig 5).

### The effects of CBN on ABR wave V are modulated by ACMS in both genotypes

**Fig 7:**
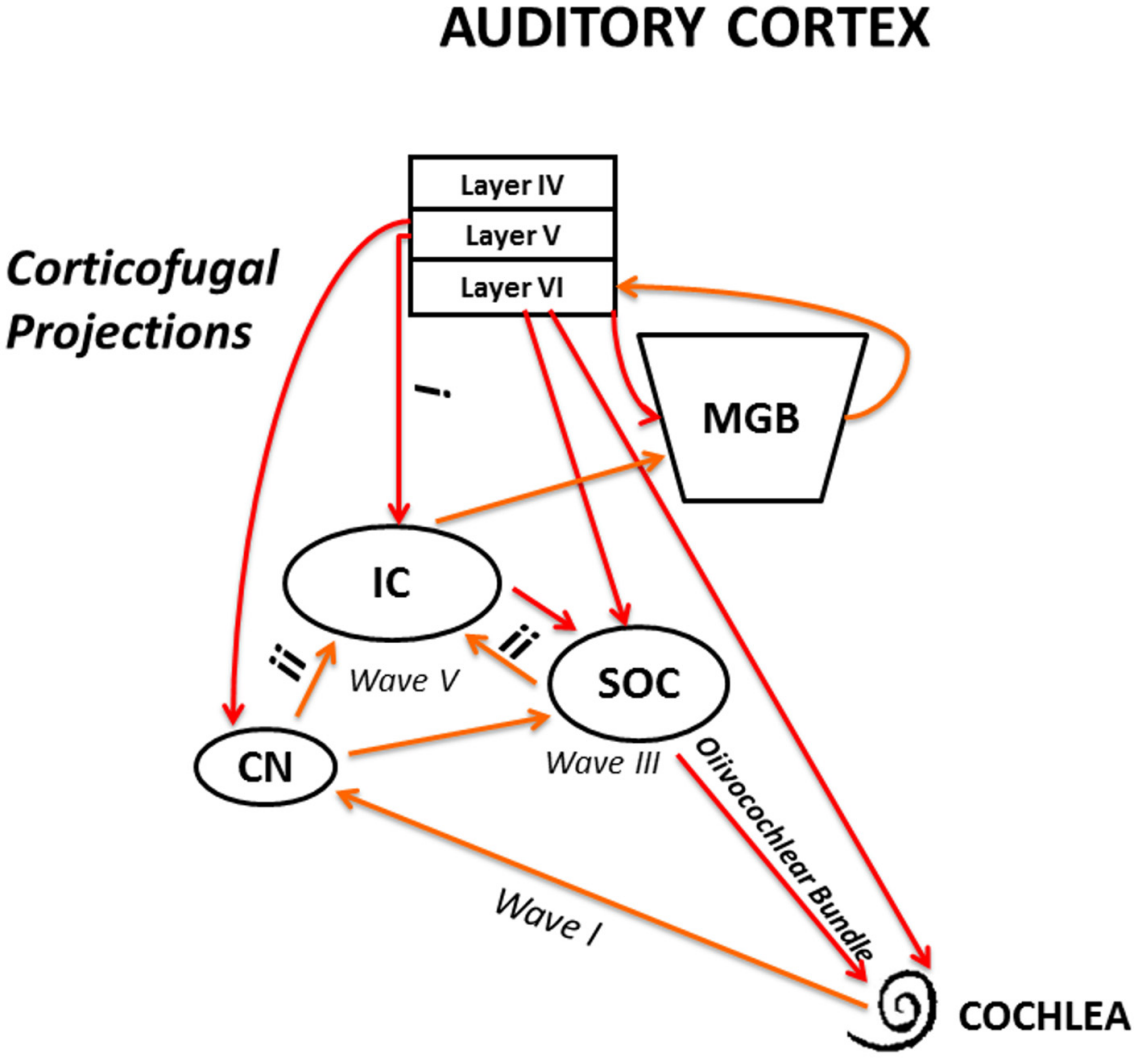
Schematic model of corticofugal effects on ABR waves. Red arrows represent descending pathways of the auditory efferent network. Orange arrows represent afferent connections of the ascending auditory pathways. From the obtained results, we propose that corticofugal effects on SOC (wave III) and auditory nerve (wave I) are driven through medial olivocochlear synapses on OHC. On the other hand, corticofugal effects on IC responses are mainly produced through direct connections (i), however indirect effects through MOC-OHC synapses, and then through ascending connections (ii) are also possible. IC: inferior colliculus; MGB: medial geniculate body; SOC: superior olivary complex; CN: cochlear nucleus.

The grand average (Fig 4) and the temporal course (Fig 6) analyses of CBN effects with ACMS on ABR wave V showed significant differences in both genotypes. However, it is important to notice that the effects were significantly larger in the WT than in the KO mice (Fig 4). It is generally accepted that ABR wave V represents responses from the inferior colliculus [35, 40]. Therefore, the present findings could be explained by the differential modulation of two different neural circuits reaching the inferior colliculus: (i) direct modulation of cortico-collicular projections or (ii) indirectly through cortico-olivocochlear connections that in turn modulate afferent responses to the inferior colliculus (Fig 7). Future work should address the possible presence of two corticofugal mechanisms (direct and indirect through MOC synapses) that modulate inferior colliculus afferent responses.

## Conclusions

Auditory cortex evoked potentials at 15 kHz were similar in WT and alpha-9-KO mice. ACMS produced significant increases of the suppressive effects of CBN on ABR waves I, III and V in WT mice, while no significant effects were obtained on ABR waves I and III in alpha-9-KO mice. These findings show that the corticofugal modulation of contralateral acoustic suppressions of auditory nerve (ABR wave I) and superior olivary complex (ABR wave III) responses are mediated through MOC synapses.

## Supporting Information

S1 File Graph data. Each worksheet contains data used in each figure.
(XLSX)
